# Jet ventilation in severe airway stenosis: how pressure and frequency shape airway pressure and ventilation

**DOI:** 10.1186/s12871-026-04075-5

**Published:** 2026-07-02

**Authors:** Mingyuan Yang, Zhuomin Deng, Hong Li, Jing Guo, Yuxue Yao, Shuwang Yang, Qinghao Cheng

**Affiliations:** 1 Department of Anesthesiology, Emergency General Hospital, Beijing, 100028 China; 2 PMLS Upstream Marketing Department, Shenzhen Mindray Bio- medical Electronics Co., LTD, Shenzhen, China

**Keywords:** Jet ventilation, Bench study, Airway geometry, Normal frequency jet ventilation, High frequency jet ventilation

## Abstract

**Background:**

Jet ventilation is widely used during rigid bronchoscopy, particularly in patients with airway narrowing, yet evidence guiding the selection of driving pressure and frequency in severely narrowed airways remains limited.

**Objectives:**

To investigate how jet driving pressure and frequency interact with airway geometry to determine airway pressure, ventilation, and gas exchange during normal-frequency jet ventilation (NFJV) and high-frequency jet ventilation (HFJV) in normal and severely narrowed airways.

**Methods:**

Jet ventilation was applied to a 3D printed adult airway model under two conditions: a normal airway and a severely narrowed airway simulating 90% fixed circumferential stenosis. Airway pressure, tidal volume, intrinsic positive end-expiratory pressure (PEEP), inspiratory and expiratory flow rates, and oxygen concentration were measured.

**Results:**

Driving pressure was the primary determinant of airway pressure, tidal volume, and inspiratory flow under both ventilation modes. Under NFJV, airway pressure increased more steeply with pressure in the normal airway, whereas under HFJV, increasing pressure led to progressive pressure accumulation in the narrowed airway, resulting in higher airway pressures at elevated settings. Intrinsic PEEP was consistently higher in the narrowed airway and was amplified by increasing pressure and frequency. Across both modes, tidal volume decreased with increasing frequency and exhibited attenuated pressure responsiveness. Although measured oxygen concentration was higher in the narrowed airway, expiratory flow was markedly reduced, indicating impaired expiratory clearance.

**Conclusions:**

Severe airway narrowing fundamentally alters the pressure-frequency-ventilation relationship during jet ventilation. In narrowed airways, escalation of driving pressure or frequency may increase pressure load and intrinsic PEEP without proportionally improving effective ventilation. Optimization should therefore prioritize adequate tidal volume, expiratory emptying, and CO_2_ monitoring rather than airway pressure or oxygen concentration alone.

**Supplementary Information:**

The online version contains supplementary material available at 10.1186/s12871-026-04075-5.

## Introduction

Jet ventilation is commonly used during rigid bronchoscopy to maintain gas exchange while allowing unobstructed access to the airway. Both normal frequency jet ventilation (NFJV) and high frequency jet ventilation (HFJV) are used in clinical practice, but optimal settings remain largely empirical, particularly in patients with critical airway narrowing [1–3].

An important feature of rigid bronchoscopy jet ventilation is that the bronchoscope sheath occupies a substantial portion of the tracheal lumen but usually does not completely seal the airway. The remaining annular space between the outer wall of the sheath and the tracheal wall provides a pathway for ambient gas entrainment and expiratory egress, and its size may therefore influence oxygen dilution, expiratory clearance, and airway pressure dissipation. In the present model, a 15-mm adult airway combined with an 11-mm rigid bronchoscope sheath produced an approximately 2-mm circumferential annular gap. This configuration was selected to approximate the limited but non-occlusive annular space encountered during adult rigid bronchoscopy rather than an arbitrarily designed airway geometry.

In stenotic airways, jet ventilation is challenged by flow limitation, impaired expiratory emptying and pressure accumulation, all of which may predispose to gas trapping, intrinsic positive end-expiratory pressure (PEEP) and hemodynamic compromise. Experimental and clinical studies suggest that these effects are amplified at higher frequencies and higher driving pressures, but the interaction between airway geometry, pressure and frequency has not been systematically quantified [4–6].

Using a controlled bench model of rigid bronchoscopy, we compared jet ventilation in a normal airway and in a severely narrowed airway. We aimed to determine how driving pressure and frequency jointly influence airway pressure, ventilation and gas exchange during NFJV and HFJV, and to identify patterns that may inform safer clinical parameter selection.

## Methods

### Study design

The experimental platform was based on a previously validated rigid bronchoscopy jet ventilation bench model [7], which was extended in the present study to incorporate a fixed severe airway stenosis. It quantified how jet driving pressure and jet frequency influence ventilatory mechanics in a normal airway versus a severely narrowed airway during jet ventilation. Ethical approval was waived for this study. The study protocol was reviewed and approved as exempt by the Academic Committee of the institution (Protocol No.KY202521), chaired by Prof. Di Wu, on 17 July 2025. The study was a bench-based experimental investigation using in vitro airway models and did not involve human participants, animals, or biological specimens. The experimental study was conducted between September 2025 and October 2025.

### Airway models

Two 3D-printed adult airway conditions were tested: (i) a normal main airway (internal diameter 15 mm; length 12 cm), and (ii) a fixed severe stenosis model (90% circumferential narrowing). In the stenosis model, the narrowed segment was ring-shaped, 1 cm in length, and located at the midpoint of the main airway; the proximal and distal segments retained the normal airway dimensions.

### Experimental setup

**Fig. 1:**
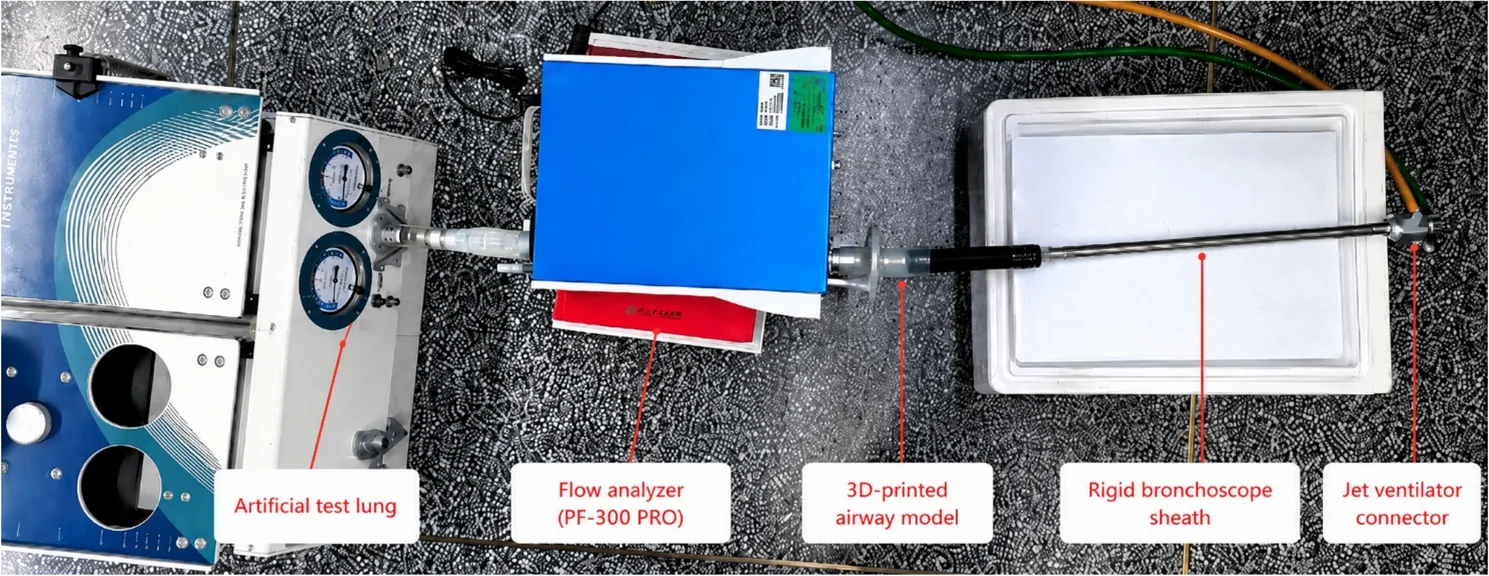
Physical laboratory setup of the rigid bronchoscopy jet ventilation bench model. The physical laboratory setup included an artificial test lung, a flow analyzer (PF-300 PRO), a 3D-printed airway model, a rigid bronchoscope sheath, and a jet ventilator connector. The jet ventilator was connected to the rigid bronchoscope sheath through the jet ventilator connector. The bronchoscope sheath was inserted into the proximal end of the 3D-printed airway model, and the distal end of the model was connected to the flow analyzer and artificial test lung. Airway pressure, flow, and oxygen concentration were measured at the distal airway, immediately upstream of the artificial test lung

The experimental circuit consisted of a jet ventilator connected to a rigid bronchoscope sheath (size 10#, Karl Storz, Germany), the 3D-printed airway model, a flow analyzer (PF-300 PRO, IMT Analytics, Switzerland), and an artificial test lung (Michigan Instruments, USA). Ventilation was provided using a Mindray A7 anesthesia workstation (Mindray A7, Shenzhen, China). The physical laboratory setup is shown in Fig. 1. The jet ventilator was connected to the rigid bronchoscope sheath through a jet ventilator connector, and the bronchoscope sheath was inserted into the proximal end of the 3D-printed airway model. The distal end of the airway model was connected in series to the flow analyzer and then to the artificial test lung. This arrangement created an open rigid-bronchoscopy jet ventilation system in which gas entered through the bronchoscope sheath and exited through the annular space between the sheath and the airway wall as well as through the downstream airway model. Airway pressure, flow, and oxygen concentration were measured at the distal airway, immediately upstream of the artificial test lung.

The rigid bronchoscope sheath was 3D-printed with an internal diameter of 10.5 mm, an external diameter of 11 mm, and a total length of 43 cm, and was designed to be compatible with a size 10# rigid bronchoscope. In both airway models, the bronchoscope tip was positioned at the same axial distance from the proximal airway opening. In the stenotic model, this position was immediately proximal to the stenotic segment; in the normal airway model, it corresponded to the same midpoint location.

The artificial test lung was configured with a capacitance of 2 L, a compliance of 50 mL·cmH₂O⁻¹, and an airway resistance of 5 cmH₂O·L⁻¹·s. These settings were kept constant throughout all experiments.

### Jet ventilation modes and parameter settings

NFJV and HFJV modes were evaluated. The inspiratory/expiratory ratio was fixed at 1:1 and the delivered oxygen fraction was set to 1.0.

Under NFJV, jet ventilation frequency ranged from 12 to 24 cycles·min⁻¹, increased in increments of 2 cycles·min⁻¹. Jet driving pressure was applied at discrete levels of 0.7, 1.1, 1.3, 1.5, 1.7, 1.9, and 2.0 bar. Under HFJV, jet ventilation frequency was set at 60, 100, 200, and 300 cycles·min⁻¹. Jet driving pressure was applied at discrete levels of 0.3, 0.5, 0.7, 0.9, 1.1, 1.3, 1.5, 1.7, 1.9, and 2.0 bar.

### Data acquisition and outcome variables

Flow and airway pressure signals were acquired at 1 kHz. After each change in mode or parameter setting, the system was allowed to stabilize for ~ 60 s before recording. For each airway condition and each pressure–frequency combination, three consecutive respiratory cycles were extracted; the full measurement was repeated three times and averaged for analysis.

Tidal volume was calculated by time integration of the flow signal over each respiratory cycle. Inspiratory and expiratory flow rates were obtained from the analyzer output; expiratory flow was recorded as negative values according to the device sign convention. Peak airway pressure and positive end-expiratory pressure (PEEP) were derived from the analyzer pressure channel at the distal airway, immediately upstream of the test lung. Oxygen fraction (FiO₂) was measured at the distal airway using the integrated oxygen measurement module of the flow analyzer, reflecting the mixed gas delivered to the test lung.

### Statistical analysis and data processing

All outcome variables including tidal volume, airway pressure, PEEP, inspiratory flow rate, expiratory flow rate, and FiO₂, were continuous and were analyzed using multivariable linear regression models. Airway type was treated as a binary categorical predictor (1 = normal airway, 0 = 90% stenosis airway), whereas jet pressure and frequency were treated as continuous predictors. Jet pressure and frequency were mean-centered prior to analysis to reduce multicollinearity and facilitate interpretation of interaction terms.

For each outcome variable, separate models were constructed for NFJV and HFJV. Initial models included only main effects, followed by inclusion of two-way interaction terms. Three-way interaction terms were evaluated but excluded if non-significant or associated with high multicollinearity. Model performance was assessed using the coefficient of determination (R²). Improvements in explanatory power were evaluated using changes in R² (ΔR²) and corresponding F-tests. Statistical significance was defined as a two-sided P value < 0.05. Three-dimensional surface plots were generated using Origin (Origin Lab, Northampton, MA, USA) to visualize the relationships between jet pressure, frequency, and outcome variables under different airway conditions.

## Results

### Airway peak pressure and intrinsic PEEP (Fig. 2a and b and Supplementary Table 1)

**Fig. 2:**
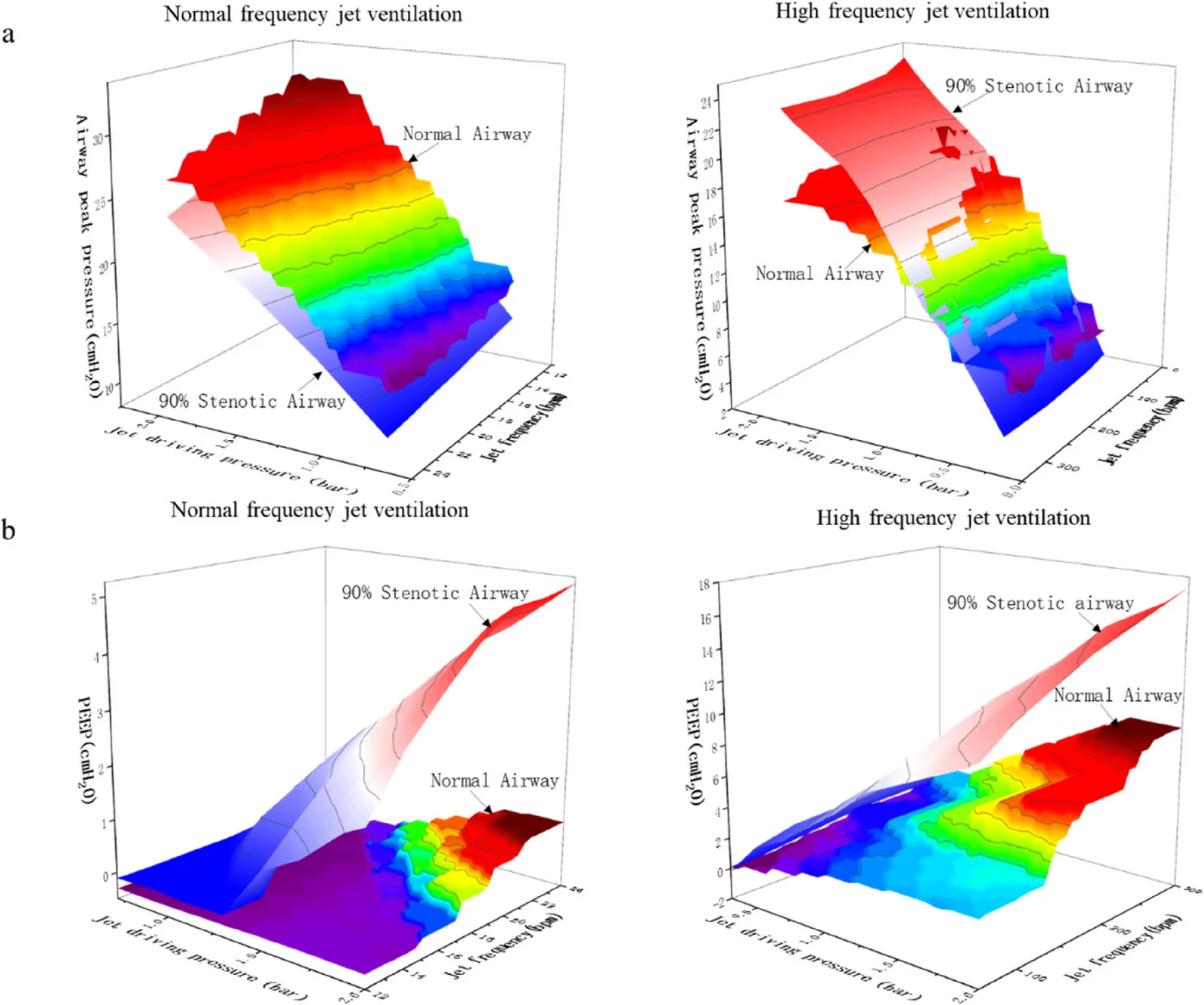
Airway peak pressure and intrinsic PEEP **a** 3D surface plot of airway peak pressure as a function of jet driving pressure and ventilation frequency during normal-frequency and high-frequency jet ventilation in normal and severely narrowed airways. **b** 3D surface plot of intrinsic positive end-expiratory pressure (PEEP) under the same conditions

#### Normal frequency jet ventilation

During NFJV, airway peak pressure increased significantly with increasing driving pressure and ventilation frequency in both airway geometries (both *P* < 0.001). The pressure-related increase in airway peak pressure was significantly steeper in the normal airway than in the stenotic airway (airway type × pressure interaction, *P* < 0.001). In addition, a smaller but statistically significant interaction between airway type and frequency was observed (*P* = 0.039), indicating that frequency-related effects differed between airway geometries. Increasing frequency attenuated the pressure-related rise in airway peak pressure (pressure × frequency interaction, *P* < 0.001).

Intrinsic PEEP increased with both driving pressure and frequency (both *P* < 0.001) and was consistently higher in the stenotic airway than in the normal airway (*P* < 0.001). The effects of pressure and frequency on intrinsic PEEP differed significantly by airway geometry (airway type × pressure and airway type × frequency interactions, both *P* < 0.001). Moreover, a significant positive pressure × frequency interaction was observed (*P* < 0.001), indicating that increases in driving pressure produced disproportionately higher intrinsic PEEP at higher frequencies.

#### High frequency jet ventilation

Under HFJV, airway peak pressure increased markedly with increasing driving pressure in both airway types (*P* < 0.001), whereas the main effect of frequency was smaller but statistically significant (*P* < 0.001). The relationship between driving pressure and airway peak pressure differed significantly by airway geometry (airway type × pressure interaction, *P* < 0.001), such that airway peak pressure increased more steeply in the stenotic airway at higher pressure settings. A significant airway type × frequency interaction was also observed (*P* = 0.004), indicating differential frequency-related effects between airway geometries.

Intrinsic PEEP increased significantly with both driving pressure and frequency (both *P* < 0.001) and remained consistently higher in the stenotic airway than in the normal airway (*P* < 0.001). The pressure-related increase in intrinsic PEEP differed significantly between airway types (airway type × pressure interaction, *P* < 0.001). In addition, a significant pressure × frequency interaction was observed (*P* < 0.001), indicating amplification of pressure-related PEEP elevation at higher frequencies. A small but statistically significant three-way interaction between airway type, pressure and frequency was observed (*P* = 0.045), reflecting differential modulation of PEEP by combined pressure and frequency effects across airway geometries.

The observed ranges and maximum values of airway peak pressure and intrinsic PEEP were further summarized across the tested pressure-frequency combinations. During NFJV, airway peak pressure ranged from 11.1 to 33.9 cmH_2_O in the normal airway and from 9.8 to 30.5 cmH_2_O in the 90% stenotic airway. The maximum airway peak pressure occurred at 2.0 bar and 12 cycles·min^− 1^ in both airway conditions. Intrinsic PEEP remained close to zero in the normal airway, with a maximum value of 0.87 cmH_2_O, whereas it increased to 5.14 cmH_2_O in the stenotic airway at 2.0 bar and 24 cycles·min^− 1^. During HFJV, airway peak pressure ranged from 3.2 to 21.6 cmH_2_O in the normal airway and from 3.0 to 24.5 cmH_2_O in the stenotic airway. The maximum airway peak pressure occurred at 2.0 bar and 60 cycles·min^− 1^. In contrast, intrinsic PEEP reached its highest values at the combination of maximum driving pressure and maximum frequency, increasing to 9.48 cmH_2_O in the normal airway and 17.41 cmH_2_O in the stenotic airway at 2.0 bar and 300 cycles·min^− 1^.

### Tidal volume and inspiratory/expiratory flow (Fig. 3a and b and Supplementary Table 2)

**Fig. 3:**
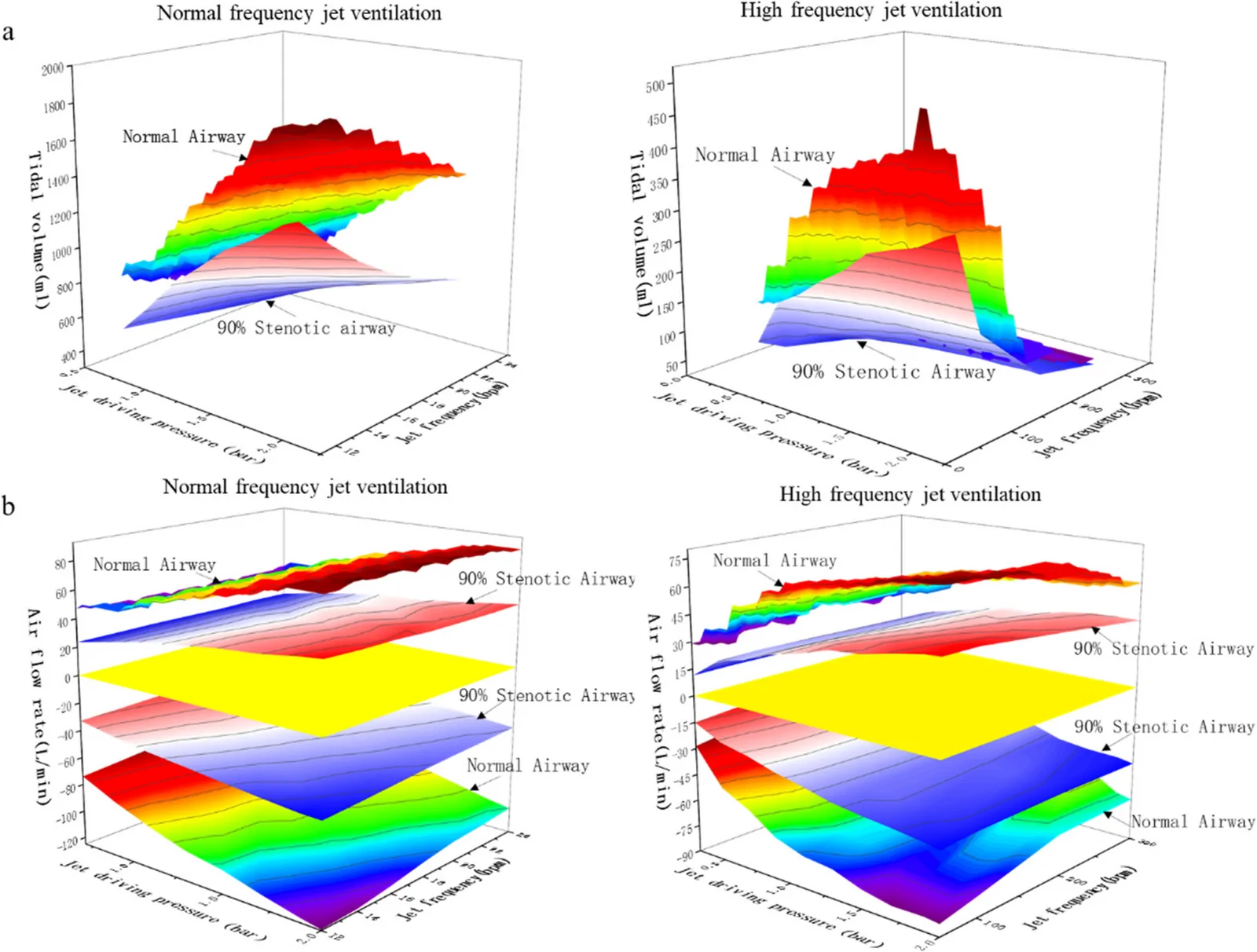
Tidal volume and inspiratory/expiratory flow **a** 3D surface plot of tidal volume as a function of jet driving pressure and ventilation frequency during normal-frequency and high-frequency jet ventilation in normal and severely narrowed airways. **b** 3D surface plot of inspiratory and expiratory flow rates under the same conditions. Note: In panel b, the upper surfaces represent inspiratory flow, the lower surfaces represent expiratory flow, and the yellow plane indicates the zero-flow reference

#### Normal frequency jet ventilation

During NFJV, tidal volume increased significantly with driving pressure and decreased with increasing frequency (both *P* < 0.001). The pressure-tidal volume relationship differed significantly between airway geometries (airway type × pressure interaction, *P* < 0.001), with attenuated pressure responsiveness in the stenotic airway. A smaller but significant airway type × frequency interaction was also observed (*P* = 0.002), indicating differential frequency-related reductions in tidal volume between airway types. Increasing frequency attenuated the pressure-related increase in tidal volume (pressure × frequency interaction, *P* < 0.001).

Under NFJV, inspiratory flow was higher in the normal airway than in the stenotic airway (*P* < 0.001 in the main-effects model) and increased with driving pressure (*P* < 0.001). After inclusion of interaction terms, the main effect of airway type was attenuated (*P* = 0.345), whereas the airway type × pressure interaction remained significant (*P* < 0.001), indicating that the pressure–inspiratory flow relationship differed between airway geometries.

Expiratory flow rate differed markedly between airway geometries, with substantially reduced expiratory flow in the stenotic airway (*P* < 0.001). Expiratory flow increased with driving pressure (*P* < 0.001) and frequency (*P* < 0.001). Significant airway type × pressure and airway type × frequency interactions were observed (both *P* < 0.001), indicating that pressure- and frequency-related effects on expiratory flow differed between airway geometries. A small but statistically significant three-way interaction was observed (*P* = 0.015).

#### High frequency jet ventilation

Under HFJV, tidal volume decreased markedly with increasing frequency (*P* < 0.001) and increased modestly with driving pressure (*P* < 0.001). Tidal volume was consistently lower in the stenotic airway than in the normal airway (*P* < 0.001). The frequency-related reduction in tidal volume differed significantly between airway types (airway type × frequency interaction, *P* < 0.001), whereas the pressure-tidal volume relationship did not differ significantly by airway geometry (airway type × pressure interaction, *P* = 0.285). Increasing frequency attenuated the pressure-related increase in tidal volume (pressure × frequency interaction, *P* < 0.001).

Under HFJV, inspiratory flow was higher in the normal airway than in the stenotic airway (*P* < 0.001) and increased with driving pressure (*P* < 0.001). Inspiratory flow decreased with increasing frequency (*P* < 0.001 in the main-effects model) and remained significant after accounting for interaction terms (*P* = 0.017 in the interaction model). Significant airway type × pressure, airway type × frequency, and pressure × frequency interactions were observed (*P* < 0.001, *P* = 0.021, and *P* = 0.001, respectively).

Expiratory flow was significantly lower in the stenotic airway than in the normal airway (*P* < 0.001). Expiratory flow increased with driving pressure (*P* < 0.001) and frequency (*P* < 0.001). Significant airway type × pressure (*P* = 0.001) and airway type × frequency interactions (*P* < 0.001) were observed, whereas the pressure × frequency interaction was not statistically significant (*P* = 0.269).

### Oxygen concentration (Supplementary Table 3)

#### Normal frequency jet ventilation

Measured oxygen concentration was significantly higher in the stenotic airway than in the normal airway (*P* < 0.001). Oxygen concentration increased with driving pressure (*P* < 0.001) and decreased with increasing frequency (*P* < 0.001). The effects of pressure and frequency on oxygen concentration differed significantly between airway geometries (airway type × pressure interaction, *P* = 0.034; airway type × frequency interaction, *P* = 0.003).

#### High frequency jet ventilation

Under HFJV, oxygen concentration remained significantly higher in the stenotic airway than in the normal airway (*P* < 0.001). Oxygen concentration increased with frequency (*P* < 0.001), whereas the main effect of pressure was not statistically significant (*P* = 0.641). However, a significant airway type × pressure interaction was observed (*P* < 0.001), indicating differential pressure-related effects on oxygen concentration between airway geometries.

## Discussion

Airway geometry emerged as a central determinant shaping the mechanical environment of jet ventilation. In the present study, the relationship between driving pressure and airway pressure differed fundamentally between ventilation modes. Under NFJV, airway pressure increased more steeply with driving pressure in the normal airway, indicating more direct pressure transmission. In contrast, under HFJV, increasing driving pressure led to progressive pressure accumulation in the narrowed airway, with airway pressure eventually exceeding that observed in the normal airway. This mode-dependent reversal may be explained by the balance between pressure transmission and expiratory pressure dissipation. During NFJV, the relatively long expiratory interval allows substantial pressure decay between cycles. In the normal airway, lower resistance permits more direct distal pressure transmission when driving pressure is increased, whereas in the stenotic airway part of the jet energy is dissipated across the narrowed segment and translated less efficiently into distal peak pressure. During HFJV, however, the markedly shortened expiratory interval limits complete pressure dissipation. The stenotic segment then behaves as a fixed flow-limiting resistor, impeding expiratory emptying and allowing residual pressure to persist between cycles. As driving pressure increases, jet kinetic energy is increasingly converted into static pressure, resulting in pressure accumulation and a steeper rise in airway peak pressure in the narrowed airway. This observation aligns with previous experimental work indicating that severe stenosis can markedly amplify proximal airway pressure during HFJV, especially with proximal jet delivery [4], and underscores how stenosis acts as a flow limiting resistor, converting jet kinetic energy into static pressure [3].

These pressure dynamics were closely coupled to expiratory constraints at the system level. Intrinsic PEEP was consistently higher in the narrowed airway and exhibited a significant positive pressure × frequency interaction, indicating that the amplifying effect of pressure on PEEP was further enhanced at higher frequencies. In stenosis settings, inadvertent high PEEP and pressure-related hazards during jet ventilation have been described experimentally and clinically, supporting the interpretation that elevated peak pressures and intrinsic PEEP may develop together when expiratory emptying is mechanically constrained [8, 9]. Clinically, complications during HFJV in interventional bronchoscopy include gas exchange derangements and pressure related adverse events, underscoring the need to treat excessive airway pressure load, including peak pressure plus intrinsic PEEP, as a central safety concern when escalation of pressure or frequency is considered [1, 3]. Although the open system used in rigid bronchoscopy may partially mitigate classic barotrauma compared with a sealed circuit [1], the present findings suggest that in the presence of severe airway narrowing, escalation of driving pressure or frequency may rapidly erode the safety margin by simultaneously increasing airway pressure and intrinsic PEEP.

Beyond pressure related safety, the present study demonstrated a dissociation between oxygenation indices and ventilatory effectiveness in narrowed airways. Although measured FiO₂ at the distal airway was consistently higher in the narrowed airway under both NFJV and HFJV, tidal volume decreased with increasing frequency and exhibited attenuated responsiveness to driving pressure. Reduced entrainment and altered mixing in stenosis models during HFJV provide a plausible explanation for higher measured FiO₂ despite constrained effective ventilation, because less ambient air is entrained and oxygen-rich gas is less diluted at the measurement site [6]. The divergent frequency-related change in measured FiO₂ between NFJV and HFJV should be interpreted as a gas-mixing phenomenon rather than as evidence of improved ventilatory efficiency. In HFJV, increasing frequency was accompanied by a marked reduction in tidal volume, indicating that effective convective volume delivery became progressively restricted at higher frequencies. Because the jet gas consisted of 100% oxygen, the concurrent increase in measured oxygen concentration most likely reflects reduced ambient air entrainment and dilution at the distal measurement site. This trend was observed in both airway geometries, suggesting that it was primarily mode-dependent. Airway narrowing further shifted the measured oxygen concentration upward, probably by additionally limiting entrainment and gas exchange with the surrounding atmosphere. Therefore, higher measured oxygen concentration during HFJV should not be interpreted as improved alveolar ventilation or CO₂ elimination.

From a physiological perspective, tidal volume and flow related indices more directly reflect ventilatory effectiveness than FiO₂ alone. Classical HFJV physiology and experimental work indicate that CO₂ elimination is strongly dependent on effective convective volume delivery and can worsen with high frequencies when incomplete exhalation and gas trapping develop [10, 11]. In the narrowed airway, increases in driving pressure in the present study preferentially augmented proximal pressure and jet momentum rather than translating proportionally into delivered volume, while higher frequencies consistently reduced tidal volume and further limited effective washout. In parallel, inspiratory flow increased with driving pressure but remained lower in the narrowed airway, whereas expiratory flow was the variable most sensitive to airway geometry and was jointly regulated by pressure and frequency, indicating that the principal ventilatory bottleneck in severe stenosis occurs during emptying rather than injection.

Clinically, impaired gas exchange remains a major limitation of jet ventilation. Hypercapnia is frequently reported during HFJV for rigid bronchoscopy, sometimes occurring despite apparently adequate oxygenation [12–14]. This dissociation suggests that oxygen-based indices alone may not reliably reflect ventilatory effectiveness in open-system jet ventilation. The risk may be further amplified in patients with airway stenosis, where expiratory emptying is mechanically constrained and pressure accumulation or gas trapping may occur [6, 15]. Although PaCO₂ was not directly measured, the convergence of reduced effective tidal volume, constrained expiratory clearance, and elevated intrinsic PEEP strongly suggests a physiological environment prone to CO₂ retention in severely narrowed airways, particularly at higher frequencies. Clinical series in rigid interventional bronchoscopy have repeatedly highlighted hypercapnia as a common complication during HFJV and emphasized the importance of CO₂ monitoring in this setting [1, 12, 13]. When frequency-related limitations dominate, strategies that restore effective volume delivery and improve expiratory clearance, such as superimposed HFJV, had been reported to improve ventilation in clinical practice and controlled comparisons, conceptually aligning with the mechanisms identified here [16, 17].

Taken together, these findings indicate that optimization of jet ventilation in severe airway narrowing should prioritize balancing driving pressure and frequency rather than maximizing either parameter in isolation. From a physiological standpoint, maintaining effective tidal volume and sufficient expiratory emptying is more critical for ventilatory adequacy than achieving higher airway pressures or FiO₂ values alone. In clinical practice, cautious pressure titration, avoidance of unnecessary high frequencies, and continuous CO₂ monitoring are likely to be essential to achieve a safer balance between oxygenation, ventilation, and airway pressure load in critically narrowed airways.

## Limitations

This study has several limitations that should be acknowledged. First, this was a bench-based experimental study using a simplified airway–lung system. Distal lung pressures, alveolar ventilation, and arterial carbon dioxide tension were not directly measured. Therefore, our interpretations are limited to system-level behavior inferred from measured airway pressure, tidal volume, flow patterns, and intrinsic PEEP. Second, only two airway conditions were examined, a normal airway and a fixed, 90% circumferential stenosis, which represents an extreme high-risk scenario. The findings should not be directly extrapolated to mild or moderate airway narrowing or to dynamic, non-uniform stenoses. Third, the analysis focused on single-mode NFJV and HFJV within predefined ranges of pressure and frequency; superimposed high frequency jet ventilation mode was not included. Finally, although the experimental setup replicates key elements of rigid bronchoscopy, including an open system and proximal jet delivery, the absence of patient-specific factors such as lung heterogeneity, active respiratory effort, and hemodynamic interactions limits direct clinical translation. These limitations notwithstanding, the controlled design allowed systematic evaluation of pressure, frequency, and airway interactions and provided mechanistic insight relevant to high-risk airway management.

## Conclusion

In this bench study, airway geometry significantly modified the effects of jet driving pressure and frequency on airway pressure and ventilation during jet ventilation. Severe airway narrowing reduced tidal volume and inspiratory and expiratory flow, increased intrinsic PEEP, and altered the relationship between driving pressure and airway peak pressure in a mode-dependent manner. Under HFJV, higher pressure and frequency settings promoted pressure accumulation in the stenotic airway. These findings suggest that, in severe airway narrowing, optimization of jet ventilation should focus on maintaining effective tidal volume and expiratory clearance while avoiding excessive pressure load.

## Supplementary Information

Supplementary Material 1.

Supplementary Material 2.

Supplementary Material 3.

## Data Availability

The datasets generated and analyzed during the current study are available from the corresponding author on reasonable request.
