# Supplementary material for: Jet ventilation in severe airway stenosis: how pressure and frequency shape airway pressure and ventilation
Source: BMC Anesthesiol. 2026 Jul 2;26:532. doi: 10.1186/s12871-026-04075-5 (PMC13520412; doi:10.1186/s12871-026-04075-5)
Supplement: Supplementary file 1 [file 12871_2026_4075_MOESM1_ESM.docx]

**Supplementary Table 3. Factors associated with oxygen concentration (Oxygen concentration is expressed as %)**

| NFJV | **Variable** | **Model 1 (Main Effects)** | | | | **Model 2 (Interaction Model)** | | | |
| --- | --- | --- | --- | --- | --- | --- | --- | --- | --- |
|  |  | **B (SE)** | **β** | **t** | ***P*** | **B (SE)** | **β** | **t** | ***P*** |
|  | **Constant** | 78.285 (0.172) | – | 454.330 | <0.001 | 78.285 (0.164) | – | 478.036 | <0.001 |
|  | **Main Effects** |  |  |  |  |  |  |  |  |
|  | Airway Type | -4.543 (0.244) | -0.804 | -18.642 | <0.001 | -4.543 (0.232) | -0.804 | -19.615 | <0.001 |
|  | Pressure | 1.220 (0.277) | 0.190 | 4.405 | <0.001 | 0.654 (0.372) | 0.102 | 1.758 | 0.082 |
|  | Frequency | -0.241 (0.030) | -0.342 | -7.927 | <0.001 | -0.153 (0.041) | -0.216 | -3.729 | <0.001 |
|  | **Interaction Effects** |  |  |  |  |  |  |  |  |
|  | Airway Type × Pressure | – | – | – | – | 1.131 (0.526) | 0.526 | 0.125 | 0.034 |
|  | Airway Type × Frequency | – | – | – | – | 0.178 (0.058) | 0.058 | -0.178 | 0.003 |
|  | Pressure × Frequency | – | – | – | – | -0.048 (0.066) | 0.066 | -0.030 | 0.463 |
|  | **Model Statistics** |  |  |  |  |  |  |  |  |
|  | R² | 0.799 |  |  |  | 0.824 |  |  |  |
|  | ΔR² | 0.794 |  |  |  | 0.814 |  |  |  |
|  | ΔR² (vs. Model 1) | – |  |  |  | 0.024 |  |  |  |
|  | F for ΔR² | – |  |  |  | 4.855 |  |  |  |
|  | P for ΔR² | – |  |  |  | 0.003 |  |  |  |
| HFJV |  | **Model 1 (Main Effects)** | | | | **Model 2 (****Interaction** **Model)** | | | |
|  |  | **B (SE)** | **β** | **t** | ***P*** | **B (SE)** | **β** | **t** | ***P*** |
|  | **Constant** | 88.065 (0.395) | – | 223.107 | <0.001 | 88.065 (0.309) | – | 284.760 | <0.001 |
|  | **Main Effects** |  |  |  |  |  |  |  |  |
|  | Airway Type | -10.834 (0.558) | -0.849 | -19.408 | <0.001 | -10.834 (0.437) | -0.849 | -24.771 | <0.001 |
|  | Pressure | 0.234 (0.499) | 0.020 | 0.469 | 0.641 | -2.933 (0.553) | 0.257 | 5.305 | <0.001 |
|  | Frequency | 0.025 (0.003) | 0.365 | 8.344 | <0.001 | 0.022 (0.003) | 0.317 | 6.531 | <0.001 |
|  | **Interaction Effects** |  |  |  |  |  |  |  |  |
|  | Airway Type × Pressure | – | – | – | – | -5.398 (0.782) | **-0.335** | -6.904 | <0.001 |
|  | Airway Type × Frequency | – | – | – | – | 0.007 (0.005) | 0.069 | 1.414 | 0.162 |
|  | Pressure × Frequency | – | – | – | – | 0.004 (0.004) | 0.037 | 1.066 | 0.290 |
|  | **Model Statistics** |  |  |  |  |  |  |  |  |
|  | R² | 0.855 |  |  |  | 0.914 |  |  |  |
|  | ΔR² | 0.849 |  |  |  | 0.907 |  |  |  |
|  | ΔR² (vs. Model 1) | – |  |  |  | 0.060 |  |  |  |
|  | F for ΔR² | – |  |  |  | 16.936 |  |  |  |
|  | P for ΔR² | – |  |  |  | <0.001 |  |  |  |

B = unstandardized coefficient; SE = standard error; β = standardized coefficient. Model 1 includes only main effects; Model 2 adds all two-way interactions. Model 3 was excluded due to a non-significant three-way interaction and high multicollinearity.

NFJV: normal frequency jet ventilation; HFJV: high frequency jet ventilation. Airway type was treated as a binary categorical predictor (1 = normal airway, 0 = 90% narrowed airway).
