# Supplementary material for: Jet ventilation in severe airway stenosis: how pressure and frequency shape airway pressure and ventilation
Source: BMC Anesthesiol. 2026 Jul 2;26:532. doi: 10.1186/s12871-026-04075-5 (PMC13520412; doi:10.1186/s12871-026-04075-5)
Supplement: Supplementary file 2 [file 12871_2026_4075_MOESM2_ESM.docx]

**Supplementary Table 1. Factors associated with airway peak pressure and intrinsic PEEP (Airway peak pressure and intrinsic PEEP are expressed in cmH_2_O.)**

| **Airway Peak Pressure** | NFJV | **Variable** | **Model 1 (Main Effects)** | | | | **Model 2 (****Interaction Model)** | | | |
| --- | --- | --- | --- | --- | --- | --- | --- | --- | --- | --- |
|  |  |  | **B (SE)** | **β** | **t** | ***P*** | **B (SE)** | **β** | **t** | ***P*** |
|  |  | **Constant** | 19.590 (0.100) | – | 196.83 | <0.001 | 19.590 (0.056) | – | 346.868 | <0.001 |
|  |  | **Main Effects** |  |  |  |  |  |  |  |  |
|  |  | Airway Type | 2.711 (0.141) | 0.221 | -19.26 | <0.001 | 2.711 (0.080) | 0.221 | 33.946 | <0.001 |
|  |  | Pressure | 13.041 (0.160) | 0.935 | 81.53 | <0.001 | 12.117 (0.128) | 0.869 | 94.402 | <0.001 |
|  |  | Frequency | -0.382 (0.018) | -0.249 | -21.74 | <0.001 | -0.404 (0.014) | -0.263 | -28.565 | <0.001 |
|  |  | **Interaction Effects** |  |  |  |  |  |  |  |  |
|  |  | Airway Type × Pressure | – | – | – | – | 1.847 (0.182) | 0.094 | 10.178 | <0.001 |
|  |  | Airway Type × Frequency | – | – | – | – | 0.042 (0.020) | 0.019 | 2.090 | 0.039 |
|  |  | Pressure × Frequency | – | – | – | – | -0.251(0.023) | -0.072 | -11.066 | <0.001 |
|  |  | **Model Statistics** |  |  |  |  |  |  |  |  |
|  |  | R² | 0.986 |  |  |  | 0.996 |  |  |  |
|  |  | ΔR² | 0.985 |  |  |  | 0.995 |  |  |  |
|  |  | ΔR² (vs. Model 1) | – |  |  |  | 0.010 |  |  |  |
|  |  | F for ΔR² | – |  |  |  | 76.81 |  |  |  |
|  |  | P for ΔR² | – |  |  |  | <0.001 |  |  |  |
|  | HFJV |  | **Model 1 (Main Effects)** | | | | **Model 2 (Interaction Model)** | | | |
|  |  |  | **B (SE)** | **β** | **t** | ***P*** | **B (SE)** | **β** | **t** | ***P*** |
|  |  | **Constant** | 14.844 (0.250) | – | 59.48 | <0.001 | 14.844 (0.204) | – | 72.713 | <0.001 |
|  |  | **Main Effects** |  |  |  |  |  |  |  |  |
|  |  | Airway Type | -0.821 (0.353) | -0.067 | -2.327 | 0.023 | -0.821 (0.289) | -0.067 | -2.845 | 0.006 |
|  |  | Pressure | 10.469 (0.315) | 0.959 | 33.184 | <0.001 | 11.858 (0.365) | 1.086 | 32.491 | <0.001 |
|  |  | Frequency | -0.007 (0.002) | -0.111 | -3.860 | <0.001 | -0.003 (0.002) | -0.042 | -1.260 | 0.212 |
|  |  | **Interaction Effects** |  |  |  |  |  |  |  |  |
|  |  | Airway Type × Pressure | – | – | – | – | -2.777 (0.516) | -0.180 | -5.381 | <0.001 |
|  |  | Airway Type × Frequency | – | – | – | – | -0.009 (0.003) | -0.098 | 2.938 | 0.004 |
|  |  | Pressure × Frequency | – | – | – | – | -0.005 (0.003) | -0.041 | -1.735 | 0.087 |
|  |  | **Model Statistics** |  |  |  |  |  |  |  |  |
|  |  | R² | 0.937 |  |  |  | 0.959 |  |  |  |
|  |  | ΔR² | 0.934 |  |  |  | 0.956 |  |  |  |
|  |  | ΔR² (vs. Model 1) | – |  |  |  | 0.023 |  |  |  |
|  |  | F for ΔR² | – |  |  |  | 13.530 |  |  |  |
|  |  | P for ΔR² | – |  |  |  | <0.001 |  |  |  |
| **PEEP** | NFJV | **Variable** | **Model 1 (Main Effects)** | | | | **Model 2 (Interaction Model)** | | | |
|  |  |  | **B (SE)** | **β** | **t** | ***P*** | **B (SE)** | **β** | **t** | ***P*** |
|  |  | **Constant** | 2.076(0.104) | – | 19.995 | <0.001 | 2.076 (0.036) | – | 57.884 | <0.001 |
|  |  | **Main Effects** |  |  |  |  |  |  |  |  |
|  |  | Airway Type | -2.164 (0.147) | -0.661 | -14.735 | <0.001 | -2.164 (0.051) | -0.661 | -42.658 | <0.001 |
|  |  | Pressure | 1.869 (0.167) | 0.502 | 11.200 | <0.001 | 3.268 (0.082) | 0.878 | 40.89 | <0.001 |
|  |  | Frequency | 0.126 (0.018) | 0.307 | 6.840 | <0.001 | 0.202 (0.009) | 0.493 | 22.503 | <0.001 |
|  |  | **Interaction Effects** |  |  |  |  |  |  |  |  |
|  |  | Airway Type × Pressure | – | – | – | – | -2.798 (0.115) | -0.532 | -24.271 | <0.001 |
|  |  | Airway Type × Frequency | – | – | – | – | -0.152 (0.013) | -0.263 | -12.021 | <0.001 |
|  |  | Pressure × Frequency | – | – | – | – | 0.118 (0.014) | 0.126 | 8.158 | <0.001 |
|  |  | **Model Statistics** |  |  |  |  |  |  |  |  |
|  |  | R² | 0.783 |  |  |  | 0.975 |  |  |  |
|  |  | ΔR² | 0.777 |  |  |  | 0.973 |  |  |  |
|  |  | ΔR² (vs. Model 1) | – |  |  |  | 0.192 |  |  |  |
|  |  | F for ΔR² | – |  |  |  | 266.718 |  |  |  |
|  |  | P for ΔR² | – |  |  |  | <0.001 |  |  |  |
|  | HFJV |  | **Model 1 (Main Effects)** | | | | **Model 3 (Full Interaction Model)** | | | |
|  |  |  | **B (SE)** | **β** | **t** | ***P*** | **B (SE)** | **β** | **t** | ***P*** |
|  |  | **Constant** | 8.067 (0.273) | – | 29.549 | <0.001 | 8.067 (0.072) | – | 112.264 | <0.001 |
|  |  | **Main Effects** |  |  |  |  |  |  |  |  |
|  |  | Airway Type | -5.050 (0.386) | -0.550 | -13.080 | <0.001 | -5.050 (0.102) | -0.550 | -49.692 | <0.001 |
|  |  | Pressure | 5.455 (0.345) | 0.664 | 15.806 | <0.001 | 7.917 (0.128) | 0.964 | 61.629 | <0.001 |
|  |  | Frequency | 0.017 (0.002) | 0.349 | 8.315 | <0.001 | 0.017 (0.001) | 0.347 | 22.166 | <0.001 |
|  |  | **Interaction Effects** |  |  |  |  |  |  |  |  |
|  |  | Airway Type × Pressure | – | – | – | – | -4.925 (0.182) | -0.424 | -27.108 | <0.001 |
|  |  | Airway Type × Frequency | – | – | – | – | 0.000 (0.001) | 0.004 | 0.243 | 0.808 |
|  |  | Pressure × Frequency | – | – | – | – | 0.015 (0.001) | 0.165 | 10.515 | <0.001 |
|  |  | Airway × Pressure × Frequency | – | – | – | – | 0.004 (0.002) | 0.032 | 2.038 | 0.045 |
|  |  | **Model Statistics** |  |  |  |  |  |  |  |  |
|  |  | R² | 0.866 |  |  |  | 0.991 |  |  |  |
|  |  | ΔR² | 0.860 |  |  |  | 0.990 |  |  |  |
|  |  | ΔR² (vs. Model 2) | – |  |  |  | 0.001 |  |  |  |
|  |  | F for ΔR² | – |  |  |  | 4.153 |  |  |  |
|  |  | P for ΔR² | – |  |  |  | 0.045 |  |  |  |

B = unstandardized coefficient; SE = standard error; β = standardized coefficient. Model 1 includes only main effects; Model 2 adds all two-way interactions. Model 3 was excluded due to a non-significant three-way interaction and high multicollinearity.

NFJV: normal frequency jet ventilation; HFJV: high frequency jet ventilation. Airway type was treated as a binary categorical predictor (1 = normal airway, 0 = 90% narrowed airway).
