# Supplementary material for: Jet ventilation in severe airway stenosis: how pressure and frequency shape airway pressure and ventilation
Source: BMC Anesthesiol. 2026 Jul 2;26:532. doi: 10.1186/s12871-026-04075-5 (PMC13520412; doi:10.1186/s12871-026-04075-5)
Supplement: Supplementary file 3 [file 12871_2026_4075_MOESM3_ESM.docx]

**Supplementary Table 2. Factors associated with tidal volume and inspiratory/expiratory flow (Tidal volume is expressed in mL; inspiratory and expiratory flow rates are expressed in L**·**min^-1^)**

| **Tidal Volume** |  | **Variable** | **Model 1 (Main Effects)** | | | | **Model 2 (Interaction Model)** | | | |
| --- | --- | --- | --- | --- | --- | --- | --- | --- | --- | --- |
|  |  |  | **B (SE)** | **Β** | **t** | ***P*** | **B (SE)** | **β** | **t** | ***P*** |
|  | NFJV | **Constant** | 773.135(14.327) | – | 53.963 | <0.001 | 773.135(4.916) | – | 157.284 | <0.001 |
|  |  | **Main Effects** |  |  |  |  |  |  |  |  |
|  |  | Airway Type | 447.034 (20.262) | 0.571 | 22.063 | <0.001 | 447.033 (6.952) | 0.571 | 64.306 | <0.001 |
|  |  | Pressure | 608.118 (23.025) | 0.683 | 26.411 | <0.001 | 415.35(11.172) | 0.467 | 31.178 | <0.001 |
|  |  | Frequency | -35.990 (2.533) | -0.368 | -14.210 | <0.001 | -38.732(1.229) | -0.396 | -31.518 | <0.001 |
|  |  | **Interaction Effects** |  |  |  |  |  |  |  |  |
|  |  | Airway Type × Pressure | – | – | – | – | 385.536 (15.799) | 0.306 | 24.402 | <0.001 |
|  |  | Airway Type × Frequency | – | – | – | – | 5.485 (1.738) | 0.040 | 3.156 | 0.002 |
|  |  | Pressure × Frequency | – | – | – | – | -28.402 (1.975) | -0.128 | -14.390 | <0.001 |
|  |  | **Model Statistics** |  |  |  |  |  |  |  |  |
|  |  | R² | 0.928 |  |  |  | 0.992 |  |  |  |
|  |  | ΔR² | 0.926 |  |  |  | 0.991 |  |  |  |
|  |  | ΔR² (vs. Model 1) | – |  |  |  | 0.064 |  |  |  |
|  |  | F for ΔR² | – |  |  |  | 270.831 |  |  |  |
|  |  | P for ΔR² | – |  |  |  | <0.001 |  |  |  |
|  | HFJV | **Constant** | 106.646 (11.579) | – | 9.210 | <0.001 | 106.646 (9.486) | – | 11.243 | <0.001 |
|  |  | **Main Effects** |  |  |  |  |  |  |  |  |
|  |  | Airway Type | 74.188 (16.376) | 0.275 | 4.530 | <0.001 | 74.188 (13.415) | 0.275 | 5.530 | <0.001 |
|  |  | Pressure | 68.958 (14.637) | 0.285 | 4.711 | <0.001 | 55.929 (16.957) | 0.232 | 3.298 | 0.001 |
|  |  | Frequency | -1.090 (0.088) | -0.751 | -12.394 | <0.001 | -0.793 (0.102) | -0.546 | -7.783 | <0.001 |
|  |  | **Interaction Effects** |  |  |  |  |  |  |  |  |
|  |  | Airway Type × Pressure | – | – | – | – | 26.059 (23.981) | 0.076 | 1.087 | 0.281 |
|  |  | Airway Type × Frequency | – | – | – | – | -0.594 (0.144) | -0.289 | -4.123 | <0.001 |
|  |  | Pressure × Frequency | – | – | – | – | -0.605(0.129) | -0.233 | -4.698 | <0.001 |
|  |  | **Model Statistics** |  |  |  |  |  |  |  |  |
|  |  | R² | 0.721 |  |  |  | 0.820 |  |  |  |
|  |  | ΔR² | 0.710 |  |  |  | 0.805 |  |  |  |
|  |  | ΔR² (vs. Model 1) | – |  |  |  | 0.099 |  |  |  |
|  |  | F for ΔR² | – |  |  |  | 13.417 |  |  |  |
|  |  | P for ΔR² | – |  |  |  | <0.001 |  |  |  |
| **Inspiratory Flow Rate** | NFJV | **Variable** | **Model 1 (Main Effects)** | | | | **Model 2 (Interaction Model)** | | | |
|  |  |  | **B (SE)** | **β** | **t** | ***P*** | **B (SE)** | **β** | **t** | ***P*** |
|  |  | **Constant** | 36.713 (0.677) | – | 54.211 | <0.001 | 36.713 (0.243) | – | 151.270 | <0.001 |
|  |  | **Main Effects** |  |  |  |  |  |  |  |  |
|  |  | Airway Type | 32.202 (0.958) | 0.782 | 33.623 | <0.001 | 32.202 (0.343) | 0.782 | 93.819 | 0.345 |
|  |  | Pressure | 26.885 (1.088) | 0.574 | 24.702 | <0.001 | 16.332(0.552) | 0.349 | 29.608 | <0.001 |
|  |  | Frequency | -0.132 (0.120) | -0.026 | -1.104 | 0.272 | -0.163 (0.061) | -0.032 | -2.681 | 0.324 |
|  |  | **Interaction Effects** |  |  |  |  |  |  |  |  |
|  |  | Airway Type × Pressure | – | – | – | – | 21.106 (0.780) | 0.319 | 27.056 | <0.001 |
|  |  | Airway Type × Frequency | – | – | – | – | 0.061 (0.086) | 0.008 | 0.712 | 0.478 |
|  |  | Pressure × Frequency | – | – | – | – | -0.179 (0.098) | -0.015 | -1.836 | 0.069 |
|  |  | **Model Statistics** |  |  |  |  |  |  |  |  |
|  |  | R² | 0.942 |  |  |  | 0.993 |  |  |  |
|  |  | ΔR² | 0.940 |  |  |  | 0.992 |  |  |  |
|  |  | ΔR² (vs. Model 1) | – |  |  |  | 0.051 |  |  |  |
|  |  | F for ΔR² | – |  |  |  | 245.302 |  |  |  |
|  |  | P for ΔR² | – |  |  |  | <0.001 |  |  |  |
|  | HFJV |  | **Model 1 (Main Effects)** | | | | **Model 2 (****Interaction Model)** | | | |
|  |  |  | **B (SE)** | **β** | **t** | ***P*** | **B (SE)** | **β** | **t** | ***P*** |
|  |  | **Constant** | 33.667 (1.138) | – | 29.584 | <0.001 | 33.667 (0.956) | – | 35.234 | <0.001 |
|  |  | **Main Effects** |  |  |  |  |  |  |  |  |
|  |  | Airway Type | 25.633 (1.609) | 0.649 | 15.927 | <0.001 | 25.633 (1.351) | 0.649 | 18.969 | <0.001 |
|  |  | Pressure | 22.682 (1.439) | 0.642 | 15.767 | <0.001 | 17.794 (1.708) | 0.504 | 10.417 | <0.001 |
|  |  | Frequency | -0.042 (0.009) | -0.200 | -4.896 | <0.001 | -0.025 (0.010) | -0.019 | -2.453 | 0.017 |
|  |  | **Interaction Effects** |  |  |  |  |  |  |  |  |
|  |  | Airway Type × Pressure | – | – | – | – | 9.774 (2.416) | 0.196 | 4.046 | <0.001 |
|  |  | Airway Type × Frequency | – | – | – | – | -0.034 (0.015) | -0.114 | -2.362 | 0.021 |
|  |  | Pressure × Frequency | – | – | – | – | -0.047 (0.013) | -0.123 | -3.586 | 0.001 |
|  |  | **Model Statistics** |  |  |  |  |  |  |  |  |
|  |  | R² | 0.874 |  |  |  | 0.915 |  |  |  |
|  |  | ΔR² | 0.869 |  |  |  | 0.908 |  |  |  |
|  |  | ΔR² (vs. Model 1) | – |  |  |  | 0.041 |  |  |  |
|  |  | F for ΔR² | – |  |  |  | 11.603 |  |  |  |
|  |  | P for ΔR² | – |  |  |  | <0.001 |  |  |  |
| **Expiratory Flow Rate** | NFJV | **Variable** | **Model 1 (Main Effects)** | | | | **Model 3 (Full Interaction Model)** | | | |
|  |  |  | **B (SE)** | **β** | **t** | ***P*** | **B (SE)** | **β** | **t** | ***P*** |
|  |  | **Constant** | -40.741 (0.687) | – | -59.287 | <0.001 | -40.741 (0.207) | – | -196.996 | <0.001 |
|  |  | **Main Effects** |  |  |  |  |  |  |  |  |
|  |  | Airway Type | -54.947 (0.972) | -0.913 | -56.539 | <0.001 | -54.947 (0.292) | -0.913 | -187.867 | <0.001 |
|  |  | Pressure | -24.085 (1.104) | -0.352 | -21.809 | <0.001 | -13.697 (0.470) | -0.200 | -29.141 | <0.001 |
|  |  | Frequency | 0.925 (0.121) | 0.123 | 7.616 | <0.001 | 0.589 (0.052) | 0.078 | 11.383 | <0.001 |
|  |  | **Interaction Effects** |  |  |  |  |  |  |  |  |
|  |  | Airway Type × Pressure | – | – | – | – | -20.776 (0.665) | -0.215 | -31.255 | <0.001 |
|  |  | Airway Type × Frequency | – | – | – | – | -0.673 (0.073) | 0.063 | 9.209 | <0.001 |
|  |  | Pressure × Frequency | – | – | – | – | 0.170 (0.118) | 0.010 | 1.448 | 0.151 |
|  |  | Airway × Pressure ×Frequency | – | – | – | – | -0.413 (0.166) | 0.017 | 2.486 | 0.015 |
|  |  | **Model Statistics** |  |  |  |  |  |  |  |  |
|  |  | R² | 0.972 |  |  |  | 0.998 |  |  |  |
|  |  | ΔR² | 0.971 |  |  |  | 0.997 |  |  |  |
|  |  | ΔR² (vs. Model 2) | – |  |  |  | 0.000 |  |  |  |
|  |  | F for ΔR² | – |  |  |  | 6.179 |  |  |  |
|  |  | P for ΔR² | – |  |  |  | 0.015 |  |  |  |
|  | HFJV |  | **Model 1 (Main Effects)** | | | | **Model 2 (Interaction** **Model)** | | | |
|  |  |  | **B (SE)** | **β** | **t** | ***P*** | **B (SE)** | **β** | **t** | ***P*** |
|  |  | **Constant** | -32.366 (0.991) | – | -32.654 | <0.001 | -32.366 (0.846) | – | -38.202 | <0.001 |
|  |  | **Main Effects** |  |  |  |  |  |  |  |  |
|  |  | Airway Type | -26.997 (1.402) | -0.707 | -19.259 | <0.001 | -26.997 (1.196) | -0.707 | -22.564 | <0.001 |
|  |  | Pressure | -20.853 (1.253) | -0.611 | -16.643 | <0.001 | -17.202 (1.512) | -0.504 | -11.374 | <0.001 |
|  |  | Frequency | 0.033 (0.008) | 0.159 | 4.342 | <0.001 | 0.005 (0.009) | 0.025 | 0.562 | 0.576 |
|  |  | **Interaction Effects** |  |  |  |  |  |  |  |  |
|  |  | Airway Type × Pressure | – | – | – | – | -7.301 (2.139) | -0.151 | -3.414 | 0.001 |
|  |  | Airway Type × Frequency | – | – | – | – | 0.055 (0.013) | 0.190 | 4.292 | <0.001 |
|  |  | Pressure × Frequency | – | – | – | – | 0.013 (0.011) | 0.035 | 1.115 | 0.269 |
|  |  | **Model Statistics** |  |  |  |  |  |  |  |  |
|  |  | R² | 0.898 |  |  |  | 0.928 |  |  |  |
|  |  | ΔR² | 0.894 |  |  |  | 0.923 |  |  |  |
|  |  | ΔR² (vs. Model 1) | – |  |  |  | 0.031 |  |  |  |
|  |  | F for ΔR² | – |  |  |  | 10.440 |  |  |  |
|  |  | P for ΔR² | – |  |  |  | <0.001 |  |  |  |

B = unstandardized coefficient; SE = standard error; β = standardized coefficient. Model 1 includes only main effects; Model 2 adds all two-way interactions. Model 3 was excluded due to a non-significant three-way interaction and high multicollinearity.

NFJV: normal frequency jet ventilation; HFJV: high frequency jet ventilation. Airway type was treated as a binary categorical predictor (1 = normal airway, 0 = 90% narrowed airway).
